# A Small Hybrid Power System of Photovoltaic Cell and Sodium Borohydride Hydrolysis-Based Fuel Cell

**DOI:** 10.3390/mi12030278

**Published:** 2021-03-07

**Authors:** Mingxue Li, Huichao Deng, Yufeng Zhang, Chenjun Hou

**Affiliations:** 1 MEMS Center, Harbin Institute of Technology, Harbin 150001, China; 16b921022@stu.hit.edu.cn (M.L.); yufeng_zhang@hit.edu.cn (Y.Z.); cjhou@hit.edu.cn (C.H.); 2 School of Mechanical Engineering and Automation, Beihang University, Beijing 100191, China

**Keywords:** NaBH_4_ hydrolysis, micro hydrogen–oxygen fuel cell system, hybrid power management

## Abstract

Although the hybrid power system that combines a photovoltaic cell and a lithium-ion battery is increasingly mature and practical, long-lifetime auxiliary power will be still needed in severe weather conditions. A small-volume hydrogen–oxygen fuel cell system based on the hydrolysis of NaBH_4_ is designed. The fuel cell system contains a tiny hydrogen generator, a hydrogen cleaner, and a small fuel cell stack consisting of three units in series. The relationship between the amount of catalyst and output performance is discussed. The long-time discharging results indicate that the fuel cell system has high power capacity. The compact design allows the fuel cell system to integrate the structure with a photovoltaic cell and lithium-ion cell and forms a hybrid power system with a small package. The power management circuit for these power sources without logic devices is designed and tested. The control strategy selects the photovoltaic–battery subsystem as the primary power source, and the fuel cell subsystem works as the backup power source to handle the circumstance when the energy stored in the battery is exhausted. The test results show that the power management system could switch the power supply automatically and timely under various emergency conditions, and the output voltage remains stable all the time.

## 1. Introduction

The wireless sensor network (WSN) is a useful network technology based on distributed wireless sensor nodes and plays an important role in many fields [1,2,3]. In particular, the node system powered by renewable sources could break through the limited working lifetime issue of a traditional battery-powered node system, which greatly enhances the practicability of the WSN [4,5]. The common environmental energy that can be acquired by the sensor system includes solar power [6,7], wind power [8,9], vibrational power [10,11], etc. However, in the field of energy harvesting, solar power generation is the most mature and reliable method with the highest efficiency [12,13]. As a matter of fact, the illumination energy is time-dependent in an actual environment, and a secondary cell such as a lithium-ion battery is introduced to enhance stability. As a result, the hybrid application of the photovoltaic cell and lithium-ion cell has attracted a lot of attention. Woerd et al. combined the photovoltaic cell and rechargeable thin-film lithium-ion battery to form a long lifetime power source for hearing aid devices and introduced a maximum-power-point-tracker (MPPT) to improve the efficiency of battery charging [14]. Xu et al. used perovskite solar cells and lithium batteries for self-charging electric vehicles and realized the storage efficiency of 7.8% [15]. Gurung et al. designed a more simple and efficiency method for the energy storage of perovskite solar cells by using a step-up converter (BOOST) and reached the overall efficiency of 9.36% [16]. Di et al. integrated dye-sensitized solar panels and a hybrid lithium battery into a small size device and realized in situ solar energy harvesting and storage, which has potential for a miniaturized wireless sensor system [17].

Although the stability of the solar cell power supply is greatly enhanced by the introduction of lithium-ion batteries, there is still an underlying danger that the energy stored in lithium-ion batteries will run out and lead to the system failing when the power system encounters a continuous lack of sunlight. As a result, adding a kind of energy storage device with high lifetime and stability as backup power for the energy system will be helpful to further improve the environmental adaptability of the energy harvesting system. As a new energy technology, the fuel cell has advantages of high capacity and high energy density [18,19]. Adopting hydrogen-rich material such as NaBH_4_ could realize the long-term stable storage of the hydrogen fuel. Utilizing NaBH_4_ as a micro hydrogen generator is an appropriate method to realize the miniaturization of the hydrogen–oxygen fuel cell system.

However, there are still some issues to be solved for the sodium borohydride fuel cell system. On the one hand, the hydrogen generation efficiency of sodium borohydride hydrolyzing could be increased by improving the catalyst materials. On the other hand, the integration of the hydrogen generator and fuel cell stack could reduce the system volume and improve the operating efficiency. The cost of the catalyst could be effectively reduced by using non-noble metals such as cobalt and nickel instead of platinum. Qiwen et al. utilized a nano nickel structure to catalyze the hydrolysis reaction of NaBH_4_ and realized the hydrogen generating rate of 4.4% [20]. Ekinci et al. synthesized a Co-La-Mi-B composite catalyst and reached the hydrogen producing rate of 9.508 L g^−1^ min^−1^, which could be used as catalyst for the fuel cell system [21]. Netskina et al. studied the difference of the nickel and cobalt catalysts and found that the nickel surface was easier to adsorb hydrogen and the reaction rate decreased under the hydrolysis condition [22]. The performance of the nickel catalyst could be enhanced by adding cobalt, which has a weak adsorption capacity for hydrogen. In addition, the structured catalyst loading method could effectively enhance the active area of the catalyst and improve the hydrogen production. Ping et al. attached the Pt/Al_2_O_3_ on the surface of large cordierite and realized the integrated structure of the catalyst and reactor which, reached a 0.45 mL/min hydrogen producing rate [23]. Tulin et al. loaded carbon nanotubes (CNT) with CoBi catalyst and investigated its performance of the NaBH_4_ hydrolysis and electrolysis [24]. The results indicated that the hydrogen production was improved. Arife et al. utilized multi-well CNTs to load Pt catalysts and reached 98% reacting efficiency, while the efficiency of commercial catalyst is 95% [25]. Mixing the catalyst with solid sodium borohydride could simplify the reactor structure. Hsueh et al. combined the sodium borohydride powder and the Co catalyst as the solid composite fuel and successfully drove a 2 W PEMFC stack to power a cell phone [26]. Netskina et al. introduced cobalt catalyst into the solid NaBH_4_ composite and found that the hydrogen-producing efficiency could be optimized by adjusting the pH value of the solution [27]. The hydrogen produced by the sodium–borohydride-based hydrogen generator could be used as the fuel of the proton exchange membrane fuel cell (PEMFC), which forms a power generating system. Thus, many researchers focus on the joint design of the hydrogen generator and fuel cell stack. Byeong et al. investigated a 500 W PEMFC power system in which hydrogen is supplied by a NaBH_4_ hydrolyzing reactor, which could work as the power source for unmanned cars and aircraft [28]. Eun et al. designed a 100 W-level sodium-borohydride-hydrolysis-based PEMFC power system for unmanned aerial vehicles (UAV) and realized stable hydrogen producing for over 3 h [29]. Kwon et al. used solid NaBH_4_ as fuel and hydrochloric acid as the medium to inject into the reactor and realized 5.1% hydrogen production, which could work for UAVs [30]. Taegyu et al. designed a high-efficiency UAV system based on the hybrid power system of a fuel cell and battery that maintained the continuous flight of the UAV for 2 h [31]. Kyunghwan et al. developed a 100 W fuel cell system used the hydrolysis of the alkaline NaBH_4_, whose catalyst is Co/Al_2_O_3_. A power management system combined with a battery was introduced to maintain the stable power supply of the UAV [32]. Jifeng et al. dispersed Co-B on the commercial polyvinyl formal (PVFM), whose hydrogen producing rate reached 200 mL/min and successfully drove a 50 W portable fuel cell [33]. Nunes et al. reported a simple structure hydrogen generator by injecting NaBH_4_ fuel. The volume of the reactor is only 9 cm^3^ and could provide hydrogen for portable fuel cell devices [34]. Lee et al. uses MEMS technology to conduct a reactor for NaBH_4_ catalytic hydrolysis, which is integrated with PEMFC. Depending on the fuel delivery by micropump, the fuel cell system reaches a 16.1 mL/min hydrogen flow rate and 174.6 mW output power [35]. Gang et al. developed a hybrid no-emission power battery for unmanned vehicles that utilized NaBH_4_-based fuel cells and a solar cell array to realize high endurance [36]. As a result, the fuel cell with an NaBH_4_-based micro hydrogen generator could realize a simple structure and small volume, which is suitable for a micro wireless sensor and handle electronic devices. However, the internal resistance of the fuel cell is higher than that of a lithium battery, and the output voltage changes obviously with the fluctuation of load current. For example, the output voltage drops to around half of the open-circuit voltage when the fuel cell works at the maximum output power [37]. The high-efficiency power converting and management is important for the fuel efficiency.

In this work, a hybrid power system that combines photovoltaic cells, a lithium-ion battery, and a micro NaBH_4_ hydrogen generator-based fuel cell subsystem is designed and tested. Aiming at this power system, a power management strategy without a microprocessor is designed to realize the power sources voltage stabilizing, energy storing, and power switching. As the power management circuit contains no logic devices such as a microprocessor, it could realize very low power consumption and fast response speed. The hybrid power system utilizes the high efficiency of the combination of the photovoltaic cell and lithium-ion battery and the high energy density of the fuel cell to realize an ultra-long lifetime for power supply, which could be applied to drive wireless sensor nodes.

## 2. Design of the Hybrid Power System

The hybrid power system utilizes the long-life and high-capacity features of the fuel cell, which works as the backup power supply for the power system to enhance the environmental adaptability of the lithium battery and photovoltaic cell structure. A small package NaBH_4_ hydrolysis-based fuel cell power generating system is integrated with the photovoltaic power system. The hybrid power system contains subsystems: a fuel cell group, hydrogen generator, photovoltaic cell, and power management module. The design structure, working principle, and implementation methods of each unit in the system are described below in detail.

### 2.1. Design of Fuel Cell Group

**Figure 1:**
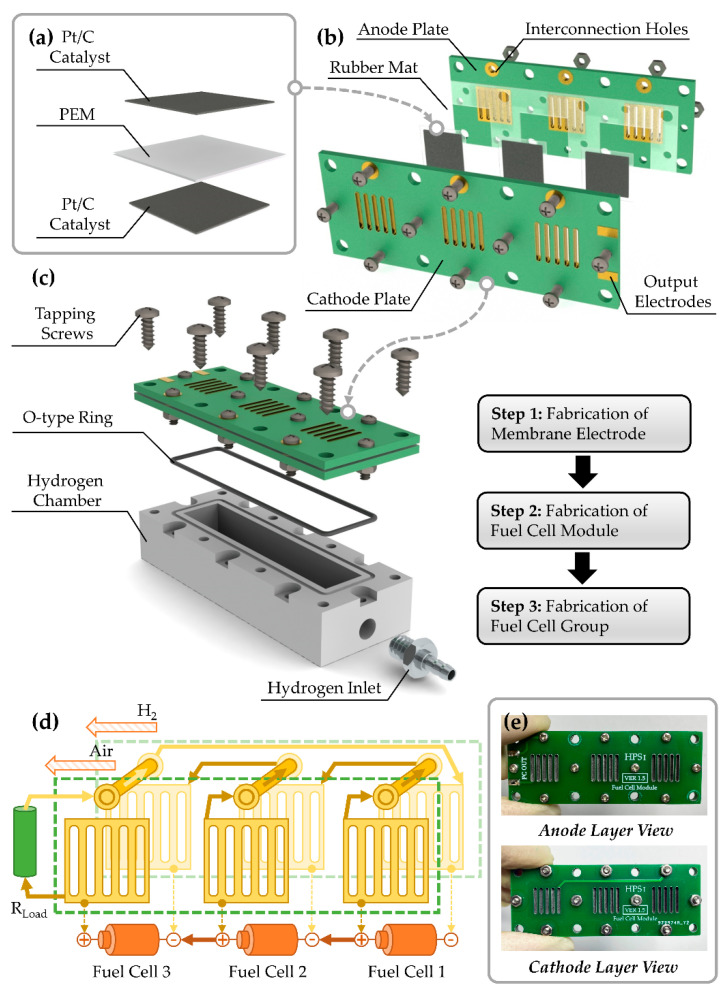
(**a**) Power generating structure of the fuel cell unit; (**b**) Assembly structure of the fuel cell module; (**c**) Assembly structure and steps of fuel cell group; (**d**) Working principle of the fuel cell group; (**e**) Photos of the fuel-cell-module prototype.

The hydrogen–oxygen fuel cell is used as the electric generating unit of the fuel cell group. The core components are the proton exchange membrane (PEM) and catalyst. The anode and cathode both adopt Pt/C as the reacting catalyst. The model of the catalyst is HISPEC-4000, which has 40% Pt with density of 2 mg/cm^2^. The size of each catalyst layer is 1.15 cm × 1.15 cm. The PEM layer is slightly larger than the catalyst layers with the size of 1.5 cm × 1.5 cm, which ensures that the catalyst of each side will not have any electrical connection during the process of packaging. The PEM thin film is Nafion 212 with the thickness of 50.8 μm. The assembly structure of the core components is shown in Figure 1a. After the catalyst layers are aligned with the center of the PEM layer, all the layers are pressed at 135 degrees Celsius for 5 min under the pressure of 15 MPa to form the fuel cell unit.

**Figure 2:**
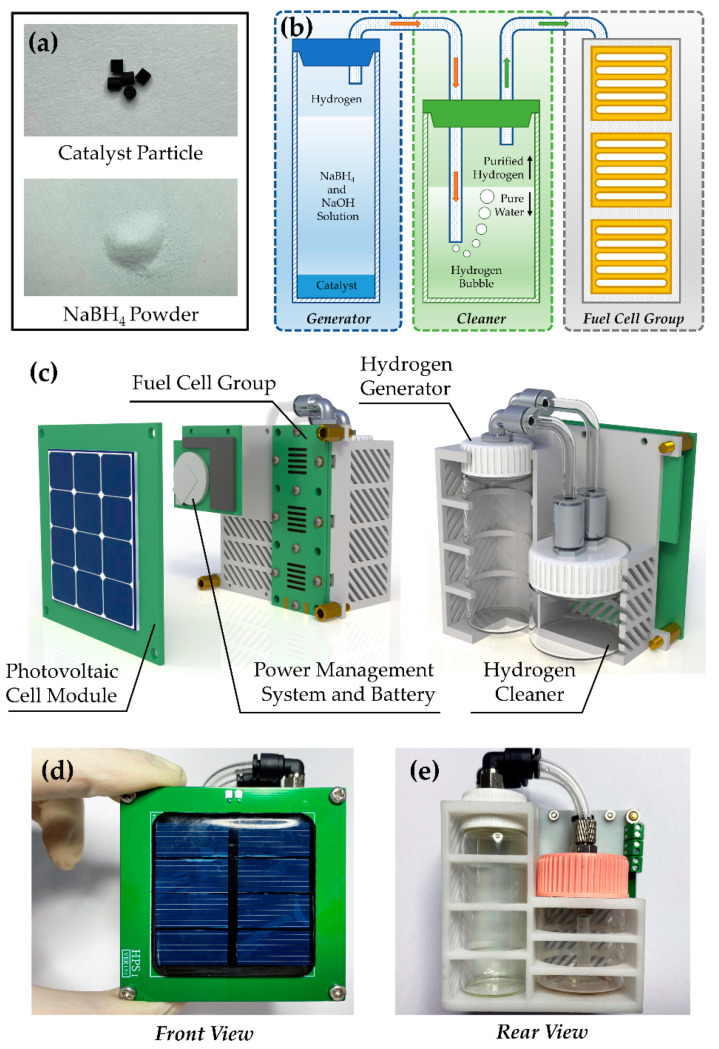
(**a**) Photos of Pt/Al_2_O_3_ catalyst particles and NaBH_4_ powder; (**b**) Working principle of the hydrogen generator; (**c**) Front view and rear view of the hybrid power system assemble; (**d**) Photos of the hybrid power system prototype (front view); (**e**) Photos of the hybrid power system prototype (rear view).

In order to obtain better output performance, three fuel cell units are used in this work to form the fuel cell group, which is shown in Figure 2b. The fuel cell group contains a cathode plate, fuel cell units, a rubber mat, and an anode plate. The cathode plate and anode plate are manufactured by a print circuit board (PCB), whose material is FR-4. This method is easy to realize and the cost is low. The collector plates of the anode and cathode can be realized by open-window patterns on the corresponding copper foil layer, and the cascade between fuel cell units is realized by the inner wires in the PCB. The electrode area in contact with the fuel cell catalyst of each unit is 1 cm × 1 cm with an aperture ratio of 42%. Thus, the PCB plates have functions of housing and electrical connection, which simplify the assembly complexity of a fuel cell group and reduce the volume. Each fuel cell unit is secured by four pairs of screws and nuts. The screws at the top provide the electrical connection between the two plates. Accordingly, the screw holes on the top of the PCB are metallized, and large open-window areas are reserved surrounding the holes to ensure that the screws and nuts can be connected into the cascading circuits reliably after locking. The screws and nuts are made of 304 L stainless steel and the model is M2. The outer contour of the rubber mat is the same as PCB plates, and the internal slots whose areas are slightly larger than the fuel cell units are reserved for fuel cell installation. In the assembly process, the rubber mat provides functions of seal and mechanical limit.

The assembled fuel cell module is fixed to the surface of the hydrogen chamber by self-tapping screws, whose model is M3 and material is 304 L stainless steel. A position-limiting groove is arranged around the hydrogen cavity for mounting the O-type rubber ring, which provides the sealing function to prevent the hydrogen leakage. The size of the hydrogen cavity is 50.8 mm × 10 mm × 8 mm, and the volume is 4.06 mL. A hydrogen inlet hole is arranged on the side of the hydrogen cavity. A stainless-steel pneumatic connector is mounted on a hydrogen cavity inlet via an M5 screw thread, which forms a hydrogen inlet with the diameter of 2.5 mm. The assembly diagram of the fuel cell group is shown in Figure 1c.

The schematic diagram of electrical connection for the fuel cell group is shown in Figure 1d. When the cathode side is exposed to air and the hydrogen chamber is filled with hydrogen, the fuel cell group starts working. The chemical reaction equations could be expressed as

(1) $$2H_{2}-4e^{-}\overset{\mathrm{Pt}}{\to }4H^{+},$$

(2) $$O_{2}+4H^{+}+4e^{-}\overset{\mathrm{Pt}}{\to }2H_{2}O.$$

Equation (1) stands for the reaction of the anode and Equation (2) stands for the reaction of the cathode. On the anode side, hydrogen loses electrons to form hydrogen ions in the presence of the Pt catalyst. The hydrogen ions form hydronium ions on the surface of PEM and pass through the PEM to the cathode. The electrons reach the cathode through external load and combine the hydrogen ions and oxygen of air to form water. As a result, a positive voltage potential difference is formed between the cathode and anode of the fuel cell, which could drive external load working. The cascade between the fuel cell units is achieved by wiring with the PCB and stainless-steel screws. The routing width of PCB is 30 mil (0.762 mm). In Figure 1d, the green dotted outlines represent the anode and cathode PCB plates, the golden rings at the top represent the metallized through holes, the top cylinders stand for the screws, and the central grids stand for the metallized collector plates. As mentioned above, the air side is the positive pole of the fuel cell unit, and the hydrogen side is the negative pole of the fuel cell unit. The positive pole of Fuel Cell 1 connects to the negative pole of Fuel Cell 2 via routes and screws, and the positive pole of Fuel Cell 2 connects to the negative pole of Fuel Cell 3. The positive pole of Fuel Cell 3 and the negative pole of Fuel Cell 1 form the output ports of the fuel cell group, and all fuel cell units are connected in series.

The prototype photos of the fuel cell module are shown in Figure 1e. The overall size is 65.6 mm × 24.8 mm × 3.6 mm, and the weight is 3.6 g.

### 2.2. Design of Hydrogen Generator

The hydrogen is the fuel of the cell group, and its simple, efficient, and secure storage is of vital importance for the hybrid power system. As a hydrogen-rich material, the NaBH_4_ has high chemical stability. In particular, the rate of natural hydrolysis is very slow when prepared as the fuel solution. Therefore, the NaBH_4_ material is suitable for a hydrogen generation system in micro or miniature hydrogen–oxygen fuel cells. As the backup power source for the power system, higher stability is required for fuel cell subsystem based on sodium borohydride hydrolysis. Although some non-precious metals, such as nickel and cobalt, could also be used as hydrolysis catalysts, the platinum is selected as the catalyst in this work, considering its excellent stability. The catalyst material is the Al_2_O_3_ particle carrying Pt (Alfa Aesar, 089106), whose diameter is around 3 mm. The catalyst could work at room temperature and is incompatible with acid, so a small amount of alkali is added to the fuel solution. The weight of a single particle is 50 mg and the content of Pt is 0.5%. The photos of the catalyst and NaBH_4_ powder are shown in Figure 2a.

The working-mechanism diagram of an NaBH_4_-solution based hydrogen generator is shown in Figure 2b. Then, the generator mainly contains the reactor and the hydrogen cleaner. Cylindrical catalyst particles have a large contact area with the fuel solution, so higher reaction rates could be achieved with only a small amount of catalyst. The granular catalysts are laid out on the bottom of the reactor to obtain a larger active area. The fuel solution consists of NaBH_4_, whose mass fraction is 13%, and NaOH, whose mass fraction is 2%. The rest of the solution is ultra-pure water. Before the fuel cell stack starts working, the fuel solution is injected into the reactor, and the solution comes into contact with the catalyst surfaces, which starts the hydrogen producing. When the fuel solution is poured into the reactor, the reaction could be expressed as

(3) $${\mathrm{NaBH}}_{4}+4H_{2}O\overset{\mathrm{Pt}}{\to }\mathrm{NaB}{\left(\mathrm{OH}\right)}_{4}+4H_{2}↑.$$

In the process of reaction, hydrogen will be mixed with tiny amounts of alkaline water solution, which is harmful to the fuel cell units. The generated hydrogen needs to be purified before it is injected into the fuel cell group, which is realized by the way of washing. As shown in the green part of Figure 2b, the bottom of the cleaner chamber is equipped with a part of pure water. The outlet of the hydrogen generator is immersed below the water surface of the cleaner. When the hydrogen is continuously produced, the pressure of the reactor is increased, and the hydrogen, which is mixed with a small amount of alkaline solution, goes through the pure water layer of the cleaner. Due to the low solubility of hydrogen, the impurities in the hydrogen will dissolve in the water, and the pure hydrogen will be discharged into the upper part of the cleaner chamber. In the end, the purified hydrogen will go into the fuel cell stack, and it works as the anode fuel. In experiments of this work, volume of 4 mL of pure water is injected into the cleaner, and the working temperature for the whole fuel cell subsystem is 20 degrees Celsius.

The hydrolytic product of sodium borohydride is sodium borate, which is slightly soluble in water at room temperature. With the consumption of sodium borohydride and water, the reactor may become saturated with sodium borate when the fuel is running out. The precipitated sodium borate crystals will adhere to the surface of catalyst particles and gradually hinder the hydrolysis reaction. As a result, a lower concentration of sodium borohydride is adopted to reduce the effect of reaction by-products, which could dissolve as much sodium borate as possible and extend the work lifetime. As a portable and compact fuel cell system, the reactor is connected to the power system through a detachable pneumatic joint. When the fuel is exhausted and the reaction stops, the fuel can be replaced with the reactor chamber taken down. Before refilling the new fuel solution, the catalyst should be immersed in water at 60 degrees Celsius for several minutes and washed off the sodium borate on its surface. Since the reactor, the cleaner, and the fuel cell stack are easily removable, the cleaning of the fuel cell system could be completed by getting the pure water flow through each part of the pipeline and drying the remaining water.

The assembly structure of the hybrid power system is shown in Figure 2c. The diameter of the hydrogen generator is 22 mm, while the diameter of the hydrogen cleaner is 30 mm. Thus, the fuel cell group is arranged on the same side as the hydrogen generator to improve the space efficiency. The inlets and outlets of the generator and cleaner adopt quick pneumatic joints to improve the replaceable capability of each part. The material of the joints is 304 L stainless steel and the material of the generator and cleaner chambers is glass. The pipelines between the hydrogen generator, the hydrogen cleaner, and the fuel cell group are composed of rubber pipes with an outer diameter of 4 mm and an inner diameter of 2.5 mm. The power management circuit and storage battery are placed next to the fuel cell group. The photovoltaic module covers the power management subsystem and fuel cell subsystem. An air gap of around 8 mm is reserved between the fuel cell group and photovoltaic cell to ensure the air supply of the cathode. The front and back photos of the hybrid-power-system prototype are shown in Figure 2d,e, respectively. The volume of the power system is 69.1 mm × 76.6 mm × 45.3 mm.

### 2.3. Design of the Hybrid Power Management Circuit

The power system in this work contains two power generators (the photovoltaic cell and the fuel cell) and one storage device (the lithium-ion battery). The power management subsystem takes charge of the constant voltage conversion, storage, and path control of the power sources. Therefore, its efficiency and stability are very important for the hybrid power system.

**Figure 3:**
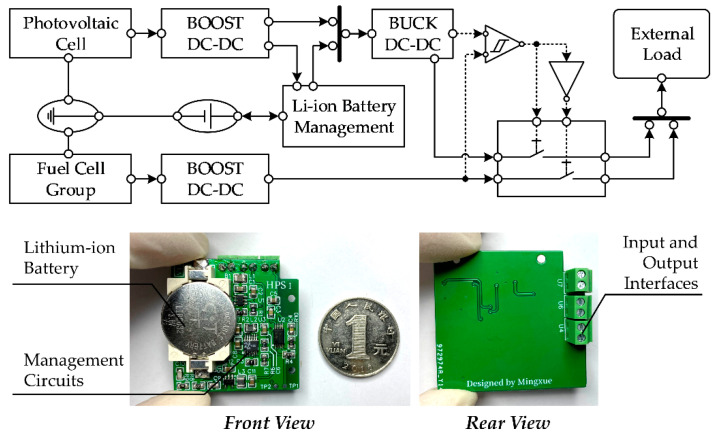
Diagram of the hybrid power management circuit and photos of the prototype with a lithium-ion battery and power management circuit (size comparison with a one RMB coin).

The structure diagram of the power management subsystem is shown in Figure 3. The output voltage of the power system is set to 3.3 V to meet the power supply requirement of most low-power devices. The photovoltaic cell is made of polysilicon, and its area is 50 mm × 50 mm with the open-circuit output circuit of 2.5 V, which is lower than the rated system voltage. The model of the lithium-ion battery paired with the photovoltaic cell is LIR-2032 with a charging voltage of 4.2 V and the rated capacity of 70 mAh. Therefore, in the first stage of photovoltaic cell input, a BOOST converter is used to stabilize the output voltage to 4.2 V. The battery charging management module contains a current regulator to maintain a constant charging current for the lithium-ion battery. The output of the lithium-ion battery is directly connected in parallel with the output of a photovoltaic cell BOOST converter through the charging management module, and it serves as the input source of the second-level BUCK voltage stabilizer, whose setting voltage is around 3.3 V. The fuel cell group could reach an open-circuit output voltage of around 2.7 V, and another BOOST converter of 3.3 V is employed. Both 3.3 V output rails of the photovoltaic cell management and the fuel cell management are accessed to the power switch array, whose on/off properties could be controlled by external signals. The control signal is generated mainly by a hysteresis comparator and an inverter. The output voltages of photovoltaic cell management and fuel cell management are compared to generate a high or low voltage level to control one of the energy paths and a synchronous inverted signal to control another path. Therefore, as long as there is an energy device available in the hybrid power system, there is always one device that could supply power for the external load. The photos of the power management subsystem are shown in Figure 3. The power management circuit and the lithium-ion battery are arranged on the same side. Despite the interfaces on the other side, all devices are surface-mounting packages, which ensures that the rear surface of the power management module could fully fit the back of the hydrogen washer chamber. The volume of the power management subsystem is slightly larger than a one RMB coin, and the area is 35.6 mm × 38.5 mm.

**Figure 4:**
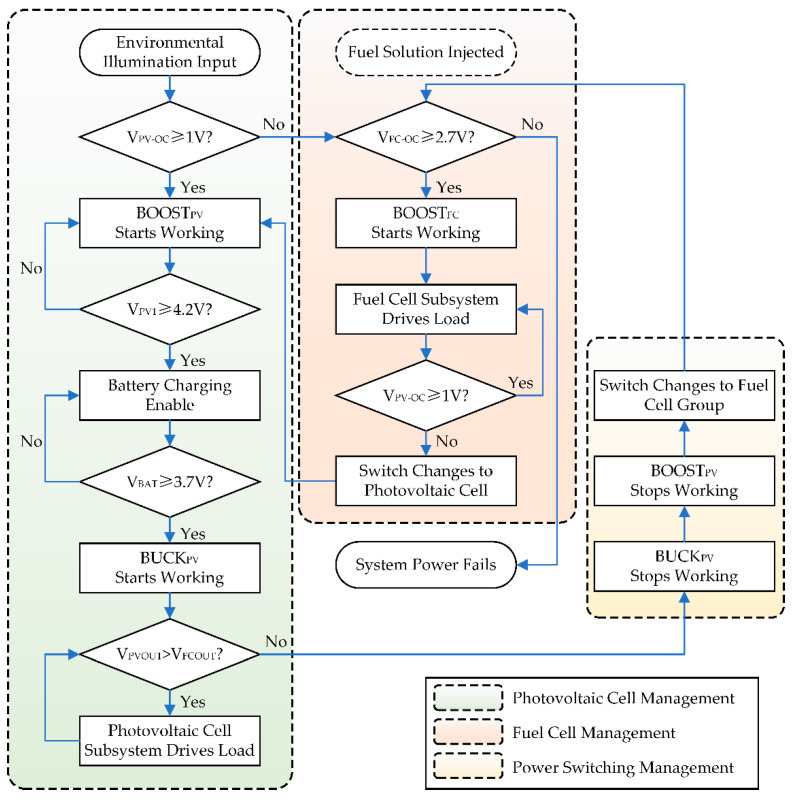
Operating flow chart of the hybrid power management system.

The working flow of the hybrid power system is shown in Figure 4, where BOOST_PV_ stands for the first-level step-up converter of the photovoltaic cell, BUCK_PV_ stands for the second-level step-down converter of the photovoltaic cell, BOOST_FC_ stands for the step-up converter of the fuel cell group, V_PV-OC_ stands for the open-circuit voltage of the photovoltaic cell, V_PV1_ stands for the output voltage of the BOOST_PV_, V_BAT_ stands for the voltage of the lithium-ion battery, V_PVOUT_ stands for the output voltage of BUCK_PV_, and V_FCOUT_ stands for the output voltage of BOOST_FC_. The overall power management strategy is based on the energy-harvesting subsystem composed of the photovoltaic cell and the lithium-ion battery as the primary source, and the fuel cell group as the secondary backup source. Thus, the working flow is divided into three sub-programs of photovoltaic cell management, fuel cell management, and power switching management. At the initial state, all the converters are disabled. When the photovoltaic cell is activated by environmental illumination, the open-circuit voltage begins generating. If the value of V_PV-OC_ exceeds the startup threshold, BOOST_PV_ begins working and pulls up V_PV1_ to 4.2 V. In the meantime, the battery charging is enabled if the battery is not fully charged, which indicates that V_BAT_ is under 4.1 V. As output ports of BOOST_PV_ and the lithium-ion battery are connected in parallel to the next level of the voltage converter, V_PV1_ will be forcefully clamped to V_BAT_ before the battery is fully charged. Therefore, when V_BAT_ is under 3.6 V, BUCK_PV_ will not have enough input voltage difference to maintain the stabilization of V_PVOUT_, and BUCK_PV_ will be enabled until V_BAT_ is charged over 3.7 V. If the photovoltaic cell fails to power the external load due to a lack of ambient light, the battery begins consuming energy stored to keep BUCK_PV_ working. The output voltage of BUCK_PV_ is slightly higher (around 0.1 V) than that of BOOST_FC_ to ensure that the photovoltaic cell subsystem has a higher priority. For the fuel cell group, when the NaBH_4_ fuel solution is injected to the reactor chamber, the fuel cell begins working. If the open-circuit voltage exceeds the threshold of 2.7 V, BOOST_FC_ converts the fuel cell voltage to 3.3 V and enters low-power standby mode. When the energy of the battery is exhausted and V_PVOUT_ falls below V_FCOUT_, the converters of the photovoltaic cell subsystem are disabled, and the power switch array enables the path of the fuel cell. The fuel cell supplies power for the external load via BOOST_FC_ and switches back to the photovoltaic cell subsystem once the voltage of the photovoltaic cell recovers. The power management strategy in this work is simple and efficient, and it could be realized only through an analog circuit instead of an additional micro-controller.

## 3. Results and Discussion

**Figure 5:**
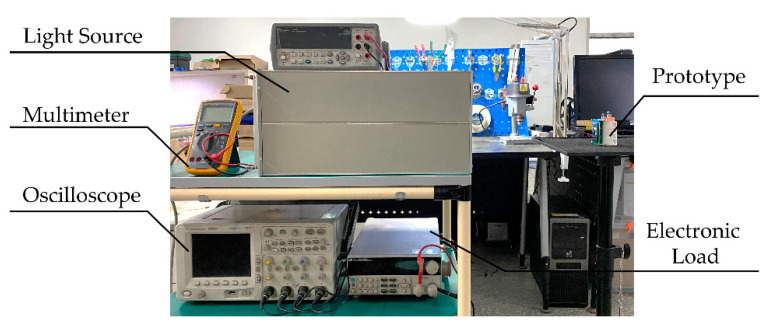
Photos of testing platform for the hybrid power system.

An electrical performance test of the hybrid power system is finished on the test platform, which includes a solar simulator, an oscilloscope, an electronic load, and digital multimeters, as shown in Figure 5. The solar simulator adopts a Xenon lamp bulb and high-voltage control circuit as a light source. The model of the Xenon lamp bulb is XHA1000 and its rated power is 1000 W. The oscilloscope is Agilent DSO-6054A, which has four sampling channels. The electronic load is ITECH IT8811 and could be programmed to scan volt–ampere characteristics. The model of the multimeters for voltage monitoring is FLUKE 18B+ with an accuracy of 4^1/2^ bits, while the model of the multimeter for current monitoring is Agilent 34410 A with an accuracy of 6^1/2^ bits.

### 3.1. Test of the Power-Generating Devices

#### 3.1.1. Photovoltaic Cell Test

**Figure 6:**
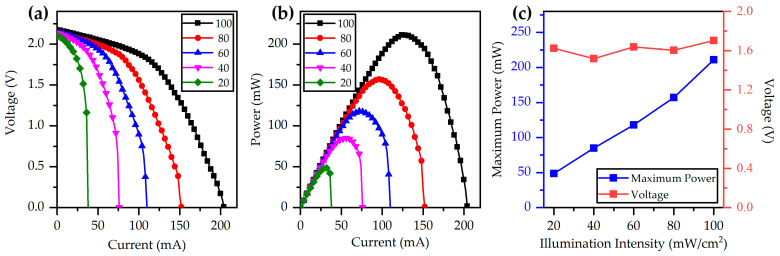
Test results of the photovoltaic cell: (**a**) The current–voltage features under different illumination intensity (the units of the legend is mW/cm^2^); (**b**) The current–power features under different illumination intensity (the units of the legend is mW/cm^2^); (**c**) The maximum output power point and corresponding voltage under different illumination intensity.

The volt–ampere features of the photovoltaic cells are tested by the electronic load, which is controlled by the computer. The test mode of the electronic load is constant current (CC), and the output voltages of each controlling current are recorded by the computer automatically. The current scanning step is 2 mA. The parameters for evaluating the intensity of ambient light are optical power density and brightness, which could be measured by an optical power meter and illuminometer, respectively. By adjusting the distance between the photovoltaic cell and light source, the output performance of the photovoltaic cell under different illumination intensity is tested, whose results are shown in Figure 6a,b. With the increasing of light power, the open-circuit voltage increases slightly. The average open-circuit voltage is 2.14 V. The output voltage decreases gradually as the load current increases, and a short-circuit current appears when the voltage drops to 0 V. The higher illumination intensity input to the photovoltaic cell, the slower the output voltage drops and the larger the reach of the short current. The output power increases first and then decreases with the increase of load current, which indicates that the maximum power point exists. Test results of the maximum power and the corresponding voltage when the photovoltaic cell is under different illumination intensity are shown in Figure 6c. With the increase of illumination intensity, the maximum output power increases approximately linearly, while the voltage fluctuates slightly in the range of 1.5 to 1.7 V. When is input light power is 20, 40, 60, 80, and 100 mW/cm^2^, the maximum output power of the photovoltaic cell is 48.7, 85.0, 117.9, 157.1, and 211.1 mW, respectively.

#### 3.1.2. Fuel Cell Test

**Figure 7:**
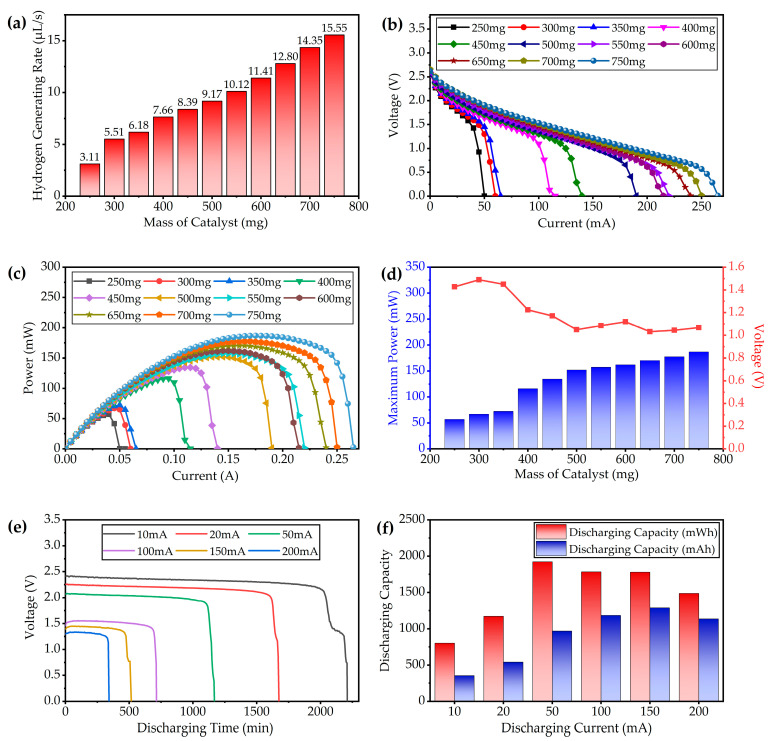
Test results of the fuel cell group: (**a**) Hydrogen production rate of the generator under different quantities of catalyst particles; (**b**) Voltage–ampere characteristic curves of the fuel cell group under different quantities of catalyst particles; (**c**) Power–ampere characteristic curves of the fuel cell group under different quantities of catalyst particles; (**d**) Maximum power points of the fuel cell group under different quantities of catalyst particles; (**e**) Long-time discharging curves of the fuel cell group under different load currents; (**f**) Discharging capacities of the fuel cell group under different discharging currents.

Due to the passive hydrogen–oxygen fuel cell structure in this work, the main factor that affects the output performance of the fuel cell is the supply of hydrogen. By adjusting the number of catalysts (particles of Al_2_O_3_ loaded with 0.5% Pt) put into NaBH_4_ fuel solution, the hydrogen-generating rate could be controlled. The rate of hydrogen generation was measured by the drainage method. In this work, a measuring cylinder with a volume of 5 mL is filled with water in advance and placed upside down in the tank where the water level is kept above the mouth of the measuring cylinder. The outlet of the hydrogen generator is connected to the inlet of the measuring cylinder via a rubber tube. When the reaction starts, the produced hydrogen is fed into the measuring cylinder. The timer starts when the hydrogen inflow reaches the 1 mL scale line, and it stops when it reaches the 4 mL scale line. Thus, the hydrogen-generating rate could be obtained by calculating the time required to produce 3 mL of hydrogen. The test results of the hydrogen-generating rate are shown in Figure 7a. With the increase of catalyst quantities, the rate of hydrogen generation increases almost linearly. When the mass of the catalyst increases from 250 mg (five particles) to 750 mg (15 particles), the rate of hydrogen generation increases from 3.11 to 15.55 µL/s. The higher rate of hydrogen production means that the fuel cell group could produce more power, but the rate at which the fuel is consumed increases as well.

The voltage–current features and the power–current features of the fuel cell are shown in Figure 7b,c, respectively. Consistent with the results of the hydrogen production test, the short-circuit current and maximum output power of the fuel cell also increase with the increase of catalyst mass. However, the open-circuit voltage maintains at around 2.6 V. With the increase of the output current, the output voltage decreases gradually. Thus, the fuel cell also has a maximum power point similar to the photovoltaic cell. The maximum power and output voltage under different amounts of catalysts are shown in Figure 7d. By adding the amounts of catalysts, the maximum power of the fuel cell could be increased, but the increase rate becomes slow when the mass of catalysts exceeds 500 mg. Considering the cost of catalyst that contains microscale precious metal Pt, the amount of catalyst could be selected appropriately according to the maximum output power demand of load in practical application.

To verify the long-time discharging capability of the fuel cell subsystem, the fuel cell undergoes an output power capacity test. The discharging current is set as a constant, and the electronic load records the output voltage of the fuel cell in real time. The amount of NaBH_4_ fuel used for the test is 15 mL. The voltage recording results are shown in Figure 7e. During the discharging process, the output voltage of the fuel cell remains almost stable, and with the gradual consumption of fuel, the output voltage drops slightly. When approaching the limit of discharging capacity, the output voltage drops rapidly, which indicates that the amount of hydrogen produced by the remaining fuel is insufficient for the hydrogen consumption under the current load current. The discharging duration grows when the load current decreases, which indicates that the fuel cell subsystem could work longer at a lower discharging time. The discharging capacity of the fuel cell could be obtained by integrating the load current and load power over time, which could be expressed as

(4) $$W_{mAh}=\int_{0}^{t_{term}}I_{load}\left(t\right)dt,$$

(5) $$W_{mWh}=\int_{0}^{t_{term}}U_{load}\left(t\right)\cdot I_{load}\left(t\right)dt,$$

where $I_{load}\left(t\right)$ is the real-time load current, $U_{load}\left(t\right)$ stands for the real-time output voltage of the fuel cell, and $t_{term}$ stands for the discharging cut-off time when the output voltage of the fuel cell drops to zero. As the load current is controlled by the electronic load, the value of $I_{load}\left(t\right)$ is considered as a constant. The test results are summarized in Figure 7f. Although the working time is longer when the fuel cell is under lower current, the capacity is lower. When the load current is 50 mA, the output capacity reaches 1.922 Wh. The current capacity comes to the maximum point of 1.288 Ah when the load current is 150 mA. At the heavy load power, the fuel cell could reach higher capacity.

According to the test results of the fuel cell, the output power is related to the number of the catalysts. The continuous working time is determined by the load current and fuel quantities. As a result, the designing amounts of catalyst and fuel should consider the type of load and expected lifetime.

### 3.2. Test of the Hybrid Power System

The power management system is responsible for the voltage stability and converting of the energy devices. In the case of power loss, the power source is actively switched by the switching logic to maintain the stability of load power supply. In addition, the battery is charged to maximize the utilization of environmental energy when the load requires less current. As a result, the output features and efficiency are the first point of concern in our power system.

**Figure 8:**
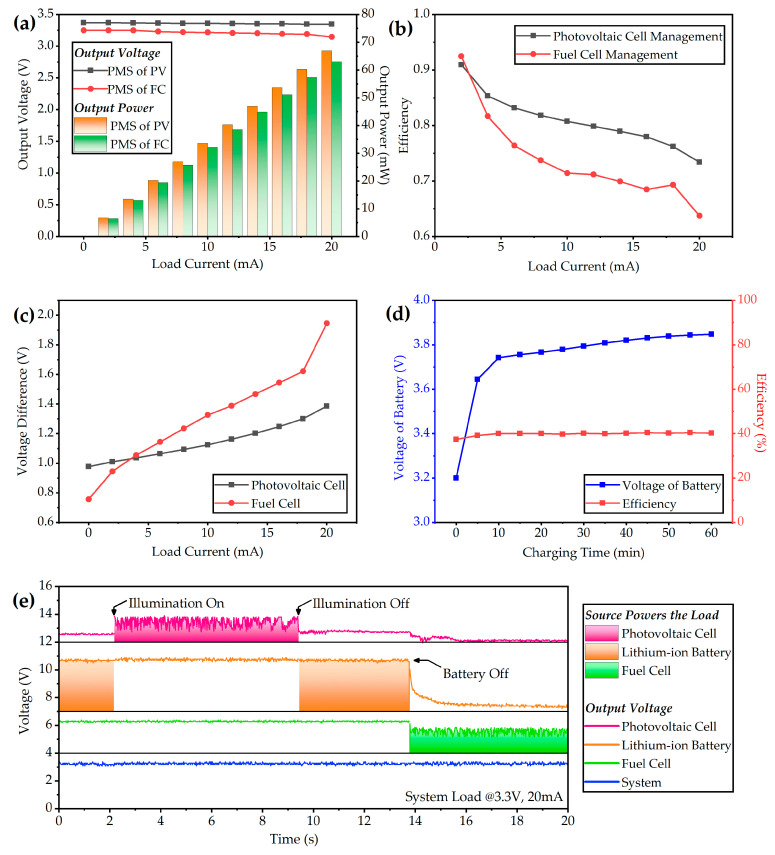
Test results of the power management system: (**a**) voltage–ampere features of the photovoltaic cell module and fuel cell module; (**b**) Power-converting efficiency under different load currents; (**c**) Difference of input voltage of the power sources and output voltage of the power management system under various currents; (**d**) Voltage of battery when charged by the photovoltaic cell and its efficiency; (**e**) Test curves of the power-switching logic when the system is under various conditions.

The hybrid power system is exposed under the illumination intensity of 50 mW/cm^2^ to simulate the sunlight, and 5 mL 13 wt % NaBH_4_ solution along with 400 mg catalyst particles are injected into the reactor. The voltage–ampere features of the power system are obtained by measuring the input and output parameters of the power source devices. The ports of the fuel cell and photovoltaic cell connect milliammeters in series and voltmeters in parallel to record the input current and voltage, respectively. The output power is monitored in real time by the electronic load. When the system is driven by a photovoltaic cell and fuel cell, the output voltage and power are shown in Figure 8a. With the increase of load current, the output voltage decreases slightly, and the voltage drop is more pronounced when the system is powered by a fuel cell. Due to the difference of output parameters at the maximum power point, the fuel cell voltage is lower and the current is higher, which results in a greater influence of internal resistance of the energy management system. The maximum drop-out voltage is 0.15 V, and the output voltage could still be considered as stable. The maximum output power of the photovoltaic cell and fuel cell is 66.94 mW and 62.90 mW, respectively. The efficiency results are shown in Figure 8b. At light load conditions, the power management efficiency could exceed 90%. With the increase of load current, the efficiency decreases. At the maximum power point, the efficiency of the photovoltaic cell and fuel cell drops to 73.4% and 63.7%, respectively. As the output power increases, either the output voltage of the fuel cell or the voltage of the photovoltaic cell will decrease, and the difference between the input voltage and output voltage increases especially for the fuel cell, which is shown in Figure 8c. The high input/output gap results in a decrease in the efficiency of BOOST regulators. At large current conditions, the voltage of the fuel cell is lower than that of the photovoltaic cell. As a result, the overall management efficiency of the power management system for the fuel cell is lower than that for the photovoltaic cell. The average management efficiency of the photovoltaic cell and fuel cell is 80.84% and 73.83%, respectively.

A small-package button-shape lithium-ion battery is integrated in the power management system. The capacity of the battery is 70 mAh and the fully charged voltage is 4.2 V. Under the illumination intensity of 50 mW/cm^2^, the lithium-ion battery is charged by the photovoltaic cell via the power management system. The voltage and charging current of the battery along with the output voltage and current are measured by amperemeters and voltage meters. The curve of the battery charging is shown in Figure 8d. The charging current is around 11.3 mA. The initial voltage of the battery is 3.2 V, and the battery is charged to 3.85 V within one hour. During this period, the accumulated energy of the lithium-ion battery is 11.43 mAh. The average energy storage efficiency is 39.85%. For the power management system, the efficiency of energy storage is lower than driving the load directly. This is because of the secondary voltage modulation during the process of the photovoltaic cell charging the battery through the power management system.

The power management strategy selects the photovoltaic cell subsystem as the primary source and the fuel cell subsystem as the backup source. When the photovoltaic cell and lithium-ion battery fail to power the load, the power management system will automatically switch the power source to the fuel cell smoothly to maintain the continuous work of the external load. In the power-switching test, various conditions that contain photovoltaic cell fails and lithium-ion battery fails are simulated by turning on or turning off the light source and removing the battery. Similar to the output characteristic test, the input catalyst mass is 400 mg, the illumination intensity is set as 50 mW/cm^2^, and the indoor lighting is around 100 Lux when the light source is turned off. The electronic load is set as constant current mode, whose value is 20 mA. During the period of the power management test, the output voltages of photovoltaic cell, fuel cell, lithium-ion battery, and external load are recorded simultaneously by the oscilloscope, and the curves are plotted in Figure 8e from top to bottom. The fountain fill stands for the load are powered by this power source. At the initial state, there is surplus energy in the battery and the light source is off, which simulates the conditions in which the illumination is lacking and the stored energy is abundant. In this state, the path from battery to load is on. When the light source is turned on, the photovoltaic cell begins powering the load, and the voltage of the battery recovers as its current drops to zero. As the switching operation of the DC-DC converter in the system, the output wave of the photovoltaic cell is fluctuant. This is to simulate the working state of the hybrid power system when the sunlight is abundant. Then, the light source is turned off, and the battery is switched to power the load again. At the last stage, the battery is removed, which simulates the condition when sunlight is insufficient and the stored energy is exhausted. The output wave of the fuel cell begins fluctuant, which indicates that the power source is switched to the fuel cell at this moment. During these power-switching operations, the output voltage of the power system remains stable with little fluctuation. It indicates that the power management system could make a fast power switching response under the circumstances of various environmental mutations, and the switching speed is fast.

## 4. Conclusions

In this work, a hybrid power system containing a photovoltaic cell, lithium-ion battery, and fuel cell is designed, fabricated, and tested. The fuel cell subsystem adopts a hydrogen–oxygen fuel cell system based on a micro NaBH_4_ hydrolyzing hydrogen generator. The designing structure of the fuel cell subsystem is simple and compact. The electricity could be generated by adding NaBH_4_ fuel solution and catalyst particles into the reactor. The output performance of the fuel cell could be adjusted by controlling the amount of catalyst. The open-circuit voltage of the fuel cell is 2.6 V and the maximum output power increases from 57.13 mW to 186.87 mW as the adding catalyst increases from 250 mg to 750 mg. Despite this, the fuel cell has high capacity, which could reach 1.92 Wh when adding 15 mL of NaBH_4_ fuel. In the actual application scene, the quantity of catalyst and fuel should be reasonably allocated, and the tradeoff between high performance and long-time discharging should be considered. According to the output features of the photovoltaic cell and fuel cell, a hybrid power management strategy is designed. The hybrid power system is mainly based on the dynamic balance between the lithium-ion battery and photovoltaic cell. When the balance is broken due to environmental change, the fuel cell works as the secondary power source to provide a longer life-extension time for the power system. For the photovoltaic cell and the fuel cell, the power management system realizes 80.84% and 73.83% average efficiency, respectively. At heavy load current that nears the maximum power point of the power devices, the large differential voltage between the input and output leads to an inefficiency in voltage stabilization. Thus, in practical application, the position of the power point under heavy load current should be controlled to improve the working voltage of fuel cell as much as possible, which could improve the efficiency of power management. The abrupt change test of the environmental light proves that the power management system could keep the output performance stable under conditions of sunlight recovering, sunlight removing, and battery exhausting. During these test conditions, the power management circuit automatically selects the proper power sources smoothly according to the designing strategy, and the response speed is fast, which ensures that no power loss for the load appears unless all the energy in the system is used up. Therefore, the long-life and high-capacity features of the fuel cell could provide long-term protection for the harsh environment of the photovoltaic cell subsystem.

However, in the process of designing and testing, we also found many deficiencies, which are also the direction of improvement in our next work. Firstly, improve the series levels of the energy devices (photovoltaic cell and fuel cell) so that the output voltage will be close to the rated voltage of the power system and the efficiency of power management could be improved. Secondly, optimize the DC–DC architecture of the power management system and reduce the efficiency loss caused by voltage stabilization with the method of adding voltage regulator reuse functions. Thirdly, improve the coupling between the hybrid power system and the load. On the one hand, the power management strategy could be dynamically adjusted by the load situation; on the other hand, the operating state of the hybrid power system could also adjust the working strategy of the load, such as the working frequency of the sensor sampling, wireless communication, etc. As briefly summarized above, in terms of hydrolytic catalysts, non-noble metal materials could be used instead of platinum to reduce the cost. The stability and working life of the reactor could be improved by optimizing the loading structure of the catalyst.
